# New biochemical pathways for forming short-chain fatty acids during fermentation in rumen bacteria*

**DOI:** 10.3168/jdsc.2023-0427

**Published:** 2023-11-04

**Authors:** Timothy J. Hackmann

**Affiliations:** Department of Animal Science, University of California, Davis, Davis, CA 95168

## Abstract

Short-chain fatty acids (SCFA) are essential to cattle as a source of energy and for other roles in metabolism. These molecules are formed during fermentation by microbes in the rumen, but even after decades of study, the biochemical pathways responsible for forming them are not always clear. Here we review recent advances in this area and their importance for improving animal productivity. Studies of bacterial genomes have pointed to unusual biochemical pathways in rumen organisms. One study found that 8% of rumen organisms forming acetate, a major SCFA, had genes for a pathway previously unknown in bacteria. The existence of this pathway was subsequently confirmed biochemically in propionibacteria. The pathway was shown to involve 2 enzymes that convert acetyl-coenzyme A to acetate. Similar studies have revealed new enzymatic steps for forming propionate and butyrate, other major SCFA. These new steps and pathways are significant for controlling fermentation. With more precise control over SCFA, cows can be fed more precisely and potentially reach higher productivity.

## Graphical Abstract

**Figure:**
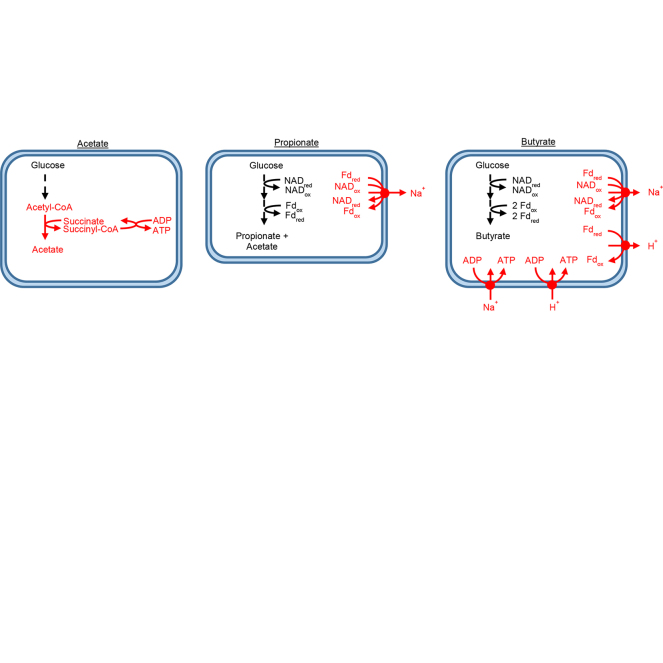

Short-chain fatty acids (**SCFA**) are crucial to the nutrition of cattle. These small molecules are formed by microbes fermenting feed in the rumen and elsewhere in the gut. They serve as a source of energy, and owing to the large quantities formed, they can account for 70% of the total energy metabolized by the host (Bergman, 1990).

Because of this importance, much study has been devoted to how SCFA are formed at the biochemical level. In rumen microbes, such study started 55 years ago when key enzymes in fermentation were first identified (Joyner and Baldwin, 1966). Despite this long history, recent discoveries point to gaps in our knowledge. This article will summarize some of these discoveries, including a new pathway for forming acetate, a major SCFA, in bacteria (Zhang et al., 2021). It will also explain the importance of these biochemical pathways in manipulating SCFA production and improving animal productivity.

Rumen microbes produce a range of SCFA during fermentation (Figure 1). Acetate, propionate, and butyrate are the most abundant, accounting for >90% of the total in the rumen by concentration (Bannink et al., 2006; Saleem et al., 2012). Many other SCFA are also formed, though in smaller amounts or under specific dietary circumstances.

**Figure 1:**
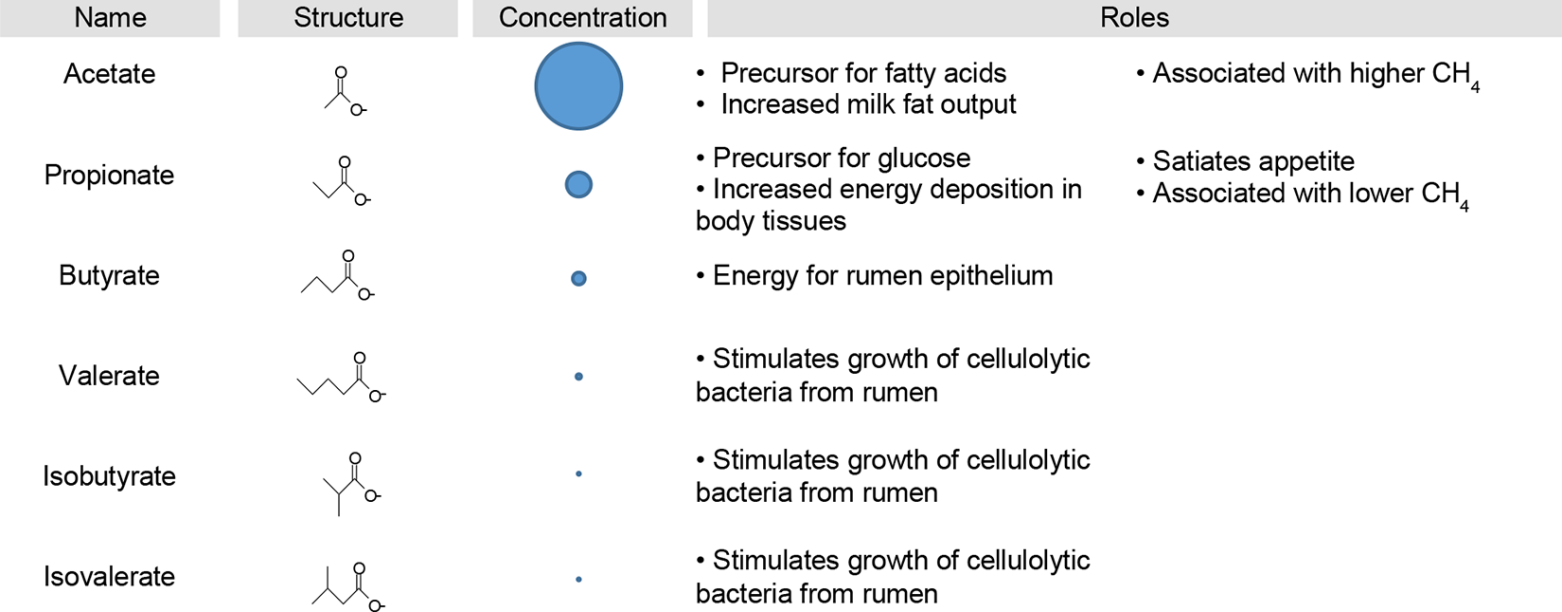
Microbes form short-chain fatty acids and other organic acids while fermenting feed in the rumen, and these acids serve a number of roles in metabolism. The top 6 (by concentration) are shown. Concentrations are from Saleem et al. (2012). References for roles for acetate are Urrutia and Harvatine (2017), Ungerfeld (2015), and Ørskov et al. (1969); for propionate are Aschenbach et al. (2010), Ørskov et al. (1969), Allen et al. (2009), and Ungerfeld (2015); for butyrate is Baldwin et al. (2004); and for valerate, isobutyrate, and isovalerate is Nagaraja et al. (1997).

Short-chain fatty acids play important roles in the host (Figure 1). One role is as a source of energy. The classic work of Bergman (1990) showed that 72% of the total energy used for maintenance metabolism of steers could be accounted by SCFA. The value for sheep was even higher (78%; Bergman, 1990). Beyond serving as a source of energy, SCFA have specific roles in metabolism (Figure 1). Acetate, for example, is a precursor for fatty acids, and infusing it in the rumen increases yield of milk fat by the cow (Urrutia and Harvatine, 2017).

Because of these roles, manipulating the production of SCFA could improve animal productivity. The manipulations could go beyond increasing acetate to increase milk fat. For example, increasing propionate would increase gluconeogenesis, decrease fatty liver and ketosis, and be valuable during transition period (see Aschenbach et al., 2010). This motivates new ways to manipulate SCFA production.

At present, there are few approaches to manipulate SCFA production precisely. One approach that seems simple at first is changing the ingredient composition of the diet. Replacing forage with concentrate ingredients, for example, increases production of propionate. This increase is observed whether production is expressed directly (mol/d; Bauman et al., 1971; Rogers and Davis, 1982a; Sutton et al., 2003) or as a molar proportion (mol/mol SCFA) (Bannink et al., 2006). A related approach is changing the chemical composition of the diet. Replacing cellulose with starch increases production of propionate, for example (Murphy et al., 1982; Bannink et al., 2006). Despite being straightforward, these approaches are not precise. Mathematical models have been developed to estimate yield of SCFA (mol/mol substrate) by chemical substrate and ingredient (Murphy et al., 1982; Bannink et al., 2006). However, models cannot explain all the observed variation in yield (Morvay et al., 2011; Nozière et al., 2011; Ghimire et al., 2014), suggesting other factors must also be important. Thus, it is hard to fine tune production of SCFA by changing ingredient and chemical composition of the diet alone.

Feed additives are another tempting approach to manipulate SCFA. Monensin, an antibiotic most effective against gram-positive bacteria (Nagaraja et al., 1997), increases production of propionate (Prange et al., 1978; Rogers and Davis, 1982b; Armentano and Young, 1983). Anti-methanogenic compounds, such as nitrate (Olijhoek et al., 2016) and fumarate (Li et al., 2018), also increase production of propionate. Other additives affecting SCFA production include buffers (Rogers and Davis, 1982b), fats (Ikwuegbu and Sutton, 1982), and plant secondary metabolites (Patra and Saxena, 2010). Additives that would affect protozoa would also change SCFA, given that rumen protozoa produce a different profile of SCFA than do bacteria (Teixeira et al., 2017). However, the changes that additives make to production are modest, inconsistent, or both. Under typical feeding rates, monensin increases propionate (mol/mol SCFA) by 10% (Ellis et al., 2012). Some plant secondary metabolites, such as saponins, are notorious for their inconsistent effects (Patra and Saxena, 2010). It would be helpful to have a more consistent and effective approach to manipulating SCFA.

A more direct approach to manipulate production of SCFA would be to engineer or enzymatically inhibit fermentation pathways. With genetic engineering, pathways forming certain SCFA could be added to or deleted from microbes. This approach has already been used to increase production of propionate in non-rumen microbes (Liu et al., 2016; Gonzalez-Garcia et al., 2020). Enzyme inhibitors could be used to target undesired pathways to similar effect. The potential of this approach is shown by 3-nitroxypropanol, an inhibitor of the terminal enzyme forming methane (Duin et al., 2016). Similar inhibitors could be developed against enzymes and pathways producing unwanted SCFA. For these approaches to be effective, the enzymes and biochemical pathways for fermentation must be known. While we have a good start in understanding biochemical pathways for fermentation, large gaps exist.

The study of biochemical pathways of fermentation has a rich history. The first pathway of fermentation was proposed by Eduard Buchner over 125 years ago (Buchner, 1897). Working with yeast, he suggested glucose was fermented directly to ethanol with a single enzyme (zymase). It soon became apparent that fermentation was more complicated, involving multiple enzymes and biochemical intermediates. Acetate, for example, was shown to be formed most immediately from the intermediate acetyl phosphate (Lipmann, 1939).

In rumen microbes, the study of fermentation at the enzymatic level began 55 years ago. Joyner and Baldwin (1966) measured catalytic activity of 21 enzymes in 9 species of rumen bacteria. The enzymes detected agreed with pathways known at the time, though there were exceptions. *Selenomonas ruminantium*, for example, appeared to lack the 2 last enzymes for forming acetate.

Complete pathways for all SCFA soon emerged and were summarized in a landmark textbook by Gottschalk (1986). One pathway, for forming acetate from acetyl-CoA, is presented in Figure 2. The pathway involves 2 steps, with acetyl phosphate as an intermediate. These pathways were delineated using a few model organisms, such as *Escherichia* *coli*, and with experiments completed over 35 years ago. Despite these limitations, most subsequent textbooks (Meganathan et al., 2007; Müller, 2008; White et al., 2012) have rendered pathways for forming SCFA as they appear in Gottschalk (1986).

**Figure 2:**
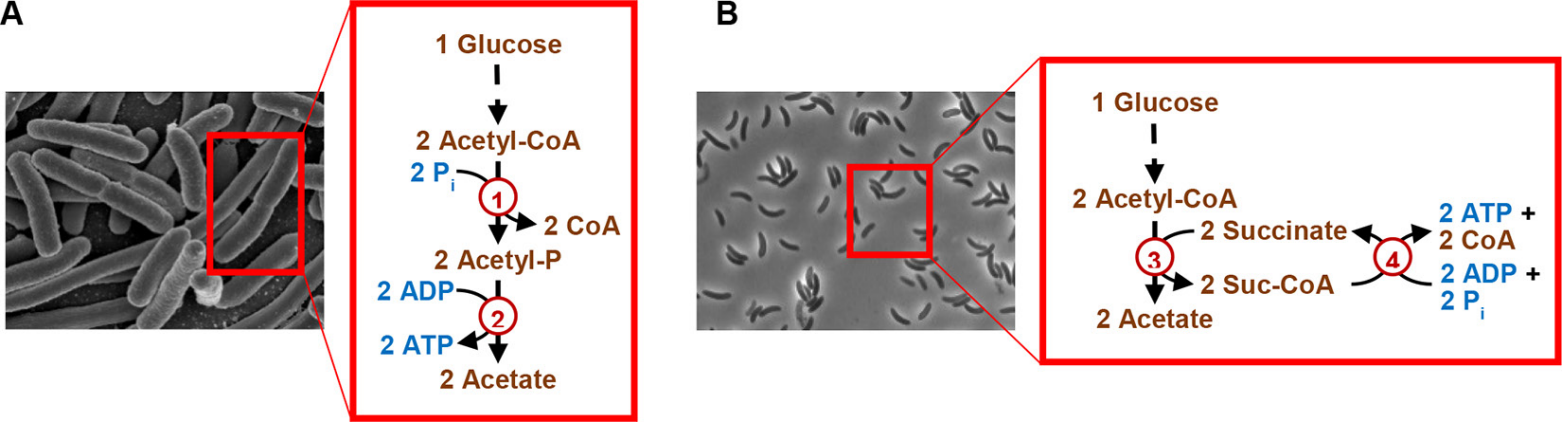
The biochemical pathway for forming acetate in (A) a landmark textbook (Gottschalk, 1986) versus (B) that encoded by genomes of certain rumen bacteria. The textbook presents a second pathway for forming acetate (from xylulose-5P), and it is not shown here. Image in (A) (of *Escherichia coli*) is from the National Institute of Allergy and Infectious Diseases (North Bethesda, MD). Image in (B) was taken by the author. Enzymes: 1, phosphotransacetylase (EC 2.3.1.8); and 2, acetate kinase (EC 2.7.2.1); 3, succinyl-CoA:acetate CoA-transferase (EC 2.8.3.18); and 4, succinyl-CoA synthetase (ADP forming; EC 6.2.1.5). P = phosphate; P_i_ = inorganic phosphate; suc-CoA = succinyl-CoA.

The first genome sequences for rumen bacteria became available about 2 decades ago (Morrison et al., 2010), and hundreds more have become available since then (Wu et al., 2009; Seshadri et al., 2018). Because enzymes for fermentation can be predicted from these sequences, this has presented a new means to examine fermentation and test the validity of textbook pathways. One surprise came with a study of the rumen bacterium *Butyrivibrio proteoclasticus* (Kelly et al., 2010). Its genome did not encode enolase, a key enzyme of glycolysis. Subsequent biochemical experiments also failed to detect significant activity (Kelly et al., 2010). This early result suggested that pathways may be more complex than previously realized.

Our laboratory subsequently examined genomes of 48 rumen bacteria (Hackmann et al., 2017). We found many did not encode enzymes of the textbook pathways. For example, 8% of bacteria observed to form acetate did not encode the usual pathway (Figure 2A). Instead, they appeared to encode a pathway previously unknown in bacteria (Figure 2, Figure 3). One of these bacteria was *S. ruminantium*, the bacterium previously found to lack enzyme activities for the usual pathway (Joyner and Baldwin, 1966). Also, we found 21% of these bacteria an unusual pathway for forming propionate or succinate and 6% had an unusual pathway for forming butyrate. Finally, we found 33% had an unusual pathway for carrying out glycolysis, such as one missing the enzyme enolase. Thus, many bacteria did not appear to follow textbook pathways of fermentation.

**Figure 3:**
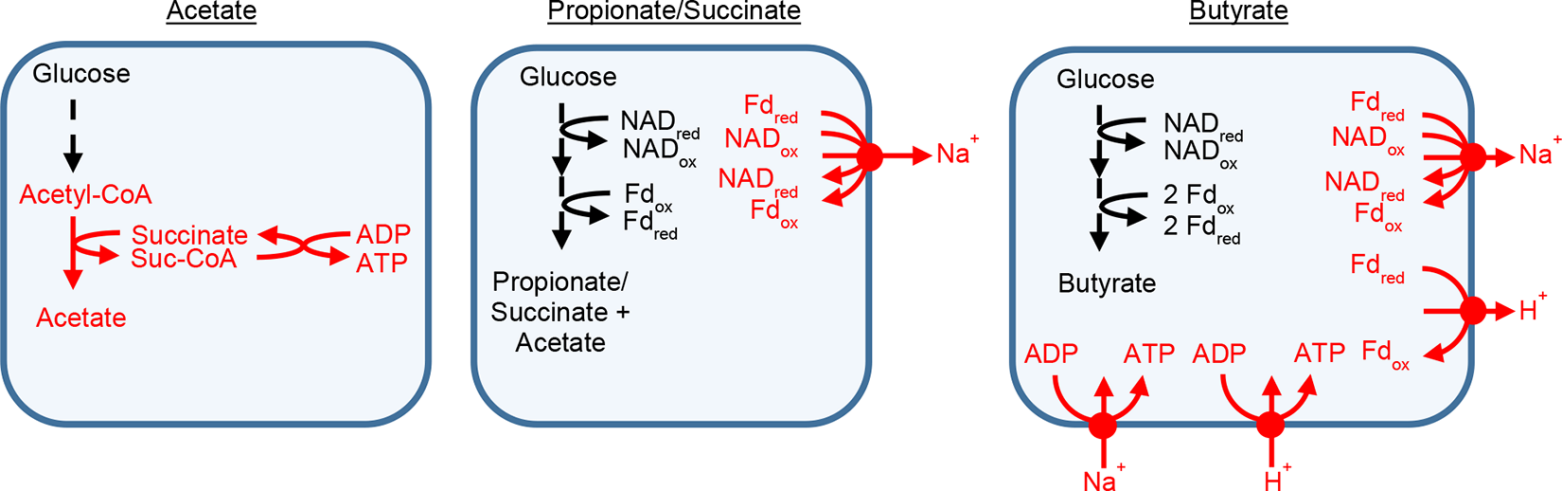
Previously unrecognized pathways or steps for forming short-chain fatty acids in bacteria. All have been confirmed biochemically in recent studies. Unrecognized steps are in red. Fd = ferredoxin; ox = oxidized; red = reduced; Suc-CoA, succinyl-CoA.

While predictions from our study were intriguing, they were ultimately hypotheses and needed experimental verification. The next step was to test the predictions from our study biochemically. Accordingly, our laboratory examined if bacteria indeed possess the pathway for forming acetate in Figure 2B (Zhang et al., 2021). This pathway had been known in a few eukaryotes (Müller et al., 2012), but it was completely unknown in bacteria. Our studies focused on *Cutibacterium granulosum* and other propionibacteria (Zhang et al., 2021). When we tested for catalytic activity of enzymes in cell extracts, we found *C*. *granulosum* indeed had the 2 expected enzymes (Figure 2, Figure 3). Further, it did not have activity of the textbook pathway (Figure 2A) or 3 other pathways previously documented in bacteria. We expressed the 2 enzymes recombinantly and found that we could reconstitute the pathway in vitro. We analyzed the AA sequence of the enzymes, and we found evolutionarily they were bacterial in origin. These findings suggested the pathway in Figure 2B is present in bacteria, representing a bacterial version of a eukaryotic pathway.

Next, we examined the pathway's distribution across bacteria from different environments. We examined genomes of n = 585 bacteria observed to form acetate, and we found 6% of them encoded genes for these enzymes (Zhang et al., 2021). This agreed well with the value (8%) we previously found for rumen bacteria (Hackmann et al., 2017). This suggests the pathway could be used widely across bacteria, not just from the rumen. Since this analysis, the pathway has been found in other bacteria, including an uncultured species (Kumar et al., 2022).

Our work on this pathway comes with a caveat. Our experiments were done with propionibacteria from human skin and other non-rumen environments. Some of these species are found in the rumen (Seshadri et al., 2018) but at low concentrations. We initially performed experiments in *S*. *ruminantium* (McCourt, 2019), a rumen bacterium that we first hypothesized had this pathway. However, we failed to detect one enzyme (succinyl-CoA:acetate CoA-transferase; EC 2.8.3.18), possibly because it was degraded to another enzyme (acetyl-CoA hydrolase; EC 3.1.2.1) that we detected instead. More work needs to be done to establish if the new pathway is indeed used in *S*. *ruminantium*, and work with isolates of propionibacteria from the rumen is ongoing in our laboratory.

Our laboratory also examined a new enzymatic step for forming propionate and succinate in bacteria (Zhang et al., 2023). When we first examined genomes of rumen bacteria forming these SCFA, we found the pathway appeared to be unbalanced (Hackmann et al., 2017). Specifically, it appeared to form excess amounts of reduced ferredoxin (**Fd_red_**) and oxidized NAD (**NAD_ox_**), which are redox cofactors. Such an unbalanced fermentation would quickly halt. We hypothesized this problem could be solved by Rnf (ferredoxin—NAD^+^ oxidoreductase [Na^+^-transporting]; EC 7.2.1.2), an enzyme that oxidizes Fd_red_ and reduces NAD_ox_ (Figure 3). This would balance fermentation and allow it to continue. This enzyme has been recognized for playing this role in other pathways, but not in forming propionate and succinate.

We tested this hypothesis in *Prevotella brevis* and *Prevotella ruminicola*, 2 predominant bacteria in the rumen (Zhang et al., 2023). Growth experiments suggested that Fd_red_ and NAD_ox_ were formed at high rates, and without Rnf, fermentation would halt within 1.5 s. Proteomics showed the cell membrane had Rnf subunits, and enzyme assays revealed the expected enzyme activity (ferredoxin—NAD[+] oxidoreductase). Thus, our experiments showed the need for Rnf and that this enzyme could fill its hypothesized role (Figure 3).

Once we established this role for Rnf in *Prevotella*, we examined its distribution across bacteria (Zhang et al., 2023). We examined n = 97 bacteria observed to form propionate, succinate, and acetate, and we found over 40% also encode Rnf. Thus, this enzyme serves a key step in the pathway of forming propionate and succinate, and it has potential to do so in many bacteria.

Butyrivibrios (*Butyrivibrio* and *Pseudobutyrivibrio*) are major butyrate-forming bacteria in the rumen, and their pathway of forming butyrate has turned out to be especially unusual. Our laboratory observed that these bacteria not only encode Rnf, the enzyme mentioned above, but also Ech (Hackmann and Firkins, 2015; Hackmann et al., 2017). These 2 enzymes both oxidize Fd_red_ and pump ions out of the cell, and due to these overlapping roles, they rarely appear in the same organism. A second laboratory subsequently found butyrivibrios encoded 2 different ATP synthases (Schoelmerich et al., 2020). One ATP synthase uses protons, and the other uses sodium ions, and again both rarely appear together. These enzymes were hypothesized to work together to balance fermentation and generate ATP (Figure 3).

Growth and biochemical experiments in *Pseudobutyrivibrio ruminis* confirm this hypothesized relationship (Schoelmerich et al., 2020). Activities of Rnf, Ech, Na^+^-dependent ATP synthase, and H^+^-dependent ATP synthase were detected in cell membranes. Further, Rnf and the Na^+^-dependent ATP synthase appeared to act in concert together, with both of their activities being stimulated by Na^+^. This suggested that the bacterium has 2 energetic circuits, one depending on Na^+^ and the other on H^+^ (Figure 3). These circuits had not been previously observed for any organism and illustrate the diversity of bacterial metabolism.

Although we have learned much about biochemical pathways for fermentation, some pathways still remain unclear. The enzyme enolase was discovered to be missing from *B*. *proteoclasticus* more than a decade ago (Kelly et al., 2010). It is still unclear how it can carry out glycolysis without it. The methylglyoxal shunt would bypass this enzyme, but this bacterium does not appear to encode it (Hackmann et al., 2017). Even if it were present, this shunt would de-energize cells, as it bypasses all ATP-producing steps of glycolysis (leading to net loss of 2 ATP/glucose). This problem is more urgent now that enolase has been found missing from the genome and proteome of more organisms (Hackmann et al., 2017; Seshadri et al., 2018; Zhang et al., 2023).

Additional areas remain poorly explored. Pathways for rumen eukaryotes (protozoa and fungi) have not received the same attention as bacteria, likely because few genome sequences for these microbes exist. Recent success in sequencing these organisms (Park et al., 2021; Li et al., 2022) will make their study easier. Pathways for minor SCFA, such as valerate (see Figure 1), also require more study. Finally, uncultured microbes (those not yet grown under laboratory conditions) represent a great unknown. Genome sequences for thousands of such organisms are now available (Stewart et al., 2019), but low genome completeness poses a challenge for determining which pathways they encode.

In summary, the biochemistry of fermentation has been studied for over 125 years, but recent discoveries revise our understanding of how all 3 major SCFA in the rumen are formed (Figure 3). The new steps and pathways forming these SCFA are logical targets for manipulating fermentation. With improved tools for genetic engineering, individual pathways in organisms can be knocked out or overexpressed. Development of enzyme inhibitors, which have been successful in reducing methane, could accomplish the same goal. These approaches would enable fine control over production of SCFA not achievable by manipulating the diet alone. Precision feeding is a major goal in ruminant nutrition, and controlling SCFA production from feed is important to achieving that precision.
